# Implementing an Online Longitudinal Leadership Development Program Using a Leadership-Specific Continuing Professional Development (CPD) Tool

**DOI:** 10.3390/pharmacy8020079

**Published:** 2020-05-06

**Authors:** Grace Oyenuga, Miranda Law, Minesh Parbat, Toyin Tofade

**Affiliations:** 1 School of Pharmacy, University of Birmingham, Birmingham B15 2TT, UK; graceoyenuga1@gmail.com; 2 Clinical and Administrative Pharmacy Sciences, Howard University College of Pharmacy, Washington, DC 20059, USA; Miranda.law@howard.edu; 3 Pharmaceutical Public Health Team, Dudley National Health Service, Clinical Commissioning Group, Brierly Hill DY5 1R2, UK; Minesh.Parbat@nhs.net; 4 Dean’s Office, Howard University College of Pharmacy, Washington, DC 20059, USA

**Keywords:** leadership development, mentorship, continuing professional development, longitudinal, online

## Abstract

As the roles of a pharmacist continue to evolve, leadership is an imperative skill for pharmacists to advance in their profession. To advance leadership behaviors, a number of tools, programs, and services have been developed worldwide to encourage the use of these behaviors in practice. A brief summary of different leadership opportunities around the globe are provided. A continuing professional development process and tool for developing and mentoring leaders that are ready to take the next step in their growth journey is introduced. This tool can be used in a live or online setting and is amenable to a longitudinal environment for leadership development and mentoring. A detailed process for implementing an online leadership development program and opportunities for future development are also described. While leadership skills can be developed in many ways, it is still unclear which methods and tools are the most effective in training pharmacists to maximize their leadership abilities. Additional research on effectiveness and impact of tools and processes for development are needed. Individuals and organizations are encouraged to consider implementing easily accessible leadership development and mentoring programs to advance the leadership skills of interested individuals.

## 1. Introduction

As the roles of a pharmacist continue to evolve, leadership is an imperative skill for pharmacists to advance in their profession. Whether the individual holds a management position or not, pharmacists must portray leadership responsibilities such as overseeing and supervising technicians or other employees, problem solving, managing change, managing finances or budgets, and making decisions regarding patient safety [1]. The main goal of this paper is to emphasize the need for leadership development around the globe and introduce a virtual leadership development program for others who may not have access to such programs by using a leadership-specific Continuing Professional Development (CPD) tool.

The definition of leadership is thought to be heavily theorized and debated. However, the traditional definition of leadership is “the action of leading a group of people or an organization” [2]. Leadership is integral for all pharmacists, regardless of position, experience and sector. Recognizing its importance, the UK’s General Pharmaceutical Council (GPhC) identified Standard 9 of the *Standards for pharmacy professionals*, as the need for pharmacists to demonstrate leadership [3]. The *Standards for pharmacy professionals* further states that pharmacists “must provide leadership to the people they work with and to others” [3]. Additionally, the Australian National Competency Standards Framework for Pharmacists presents “Leadership of self” and “Showing leadership in practice” as standards containing enabling competencies. These enabling competencies encourage pharmacists “to serve as a role model, coach and mentor for others”, as well as “inspire a strategic plan with a common purpose”, alongside colleagues [4]. Although these standards and recommendations have been published, authors White and Enright carried out a seven-year follow-up study to conclude that there is still a “leadership crisis” within the pharmacy profession [5]. While the available leadership development programs are increasing, there are still limited programs available for pharmacists worldwide. In an attempt to advance leadership competencies and behaviors, a number of tools, programs, and services have been developed worldwide to encourage the use of these behaviors in practice. An example of this is the Academic Leadership Fellows Program, organized by the American Association of Colleges of Pharmacy (AACP), which consists of four sessions that support and contribute to the development of pharmacy leaders in the academic sector [6]. Another example is the Leadership Development Framework (LDF) which is an “outline of behaviors required to become an engaging leader. “The behaviors within this framework consist of reflective questions which prompt thinking around motivation, intentions and the evaluation of behaviors surrounding leadership [7].

## 2. Leadership in the Different Regions

Opportunities for pharmacists to advance their leadership abilities are available; however, these programs seem to be few and far between. In some regions of the world, e.g., South America, there seems to be a lack of leadership development programs and tools available for pharmacists. Many of the programs or courses are not funded by a governing body, therefore, pharmacists need to pay for the program or course themselves. This may be a deterrent for pharmacists to join and be a part of a course which can develop their leadership skills. This is especially true for those who do not see the significance of leadership in personal development and the pharmacy profession. An example of this is the Top Pharmacy Leadership program which was withdrawn as an available course due to the lack of funding by the National Health Service (NHS) [8]. This emphasizes the importance of both pharmacists and governing bodies to see the value in leadership training and, most importantly, how it can improve patient care. A brief summary of select opportunities around the globe are available in Table 1.

Despite the availability of all these programs, at global conferences, there are still requests from conference participants, to engage in easily accessible programs that could be done in a virtual environment. The aim is to allow for ongoing professional development and mentorship, over a long period of time for about a year or two. We describe here a program that was developed and refined to address these requests.

## 3. Implementing a Leadership Development Framework Using a Leadership-Specific CPD Tool

In 2019, an international team of pharmacy professionals and leaders embarked upon a process to develop and mentor interested young pharmacy professionals from around the world. The goal was to build upon the key foundational leadership principles of teamwork, project management, communication, and service using a continuing professional development framework [28,29]. A leadership development CPD Tool, now referred to as the CPD for Leaders Tool, was used to guide the key steps in a CPD process of Reflect, Plan, Act and Evaluate [29]. The Tool was first piloted at the American Association for Colleges of Pharmacy (AACP) Leadership Forum in April of 2019. Leaders of the AACP special Interest groups, sections and board members were in attendance at the workshop session focusing on using a CPD process for leadership development. A facilitator worked with an audience of about 50 participants. Participants worked in groups of four or at a round table of 8–10 people.

Participants were given the CPD for Leaders tool (Supplementary S1) and asked to reflect individually on behaviors, skills, and competencies that they believe are desirable attributes of a leader. Participants were encouraged to record these leadership attributes including their desired leadership competencies in the Reflect section of the CPD for Leaders Tool. After a period of 15 min, the leaders were encouraged to share with their peers to generate further discussion at the respective tables. The facilitator has also used the following questions to generate reflective discussion to list desired attributes of a leader and assist with identifying gaps the participant may have (e.g., Think about your favorite and least favorite supervisor/boss. How did they conduct business? What do you admire most about them? What was it like to work with this individual?) After discussing with a peer, the facilitator encouraged group reflection by asking the participants to discuss their results at the table. During the table discussion, participants were expected to identify a lead or scribe so that the common themes from the individually identified desired competencies/expectations could be recorded. Furthermore, each scribe was instructed to make a list of the top five desired competencies for the table and prepare to report out to the larger group of participants at the Leadership Forum. The scribe used a large post-it paper to list the top five desired competencies at their respective tables and shared with the large group. The facilitator identified select table groups to report out their findings to the larger group. Afterwards, table groups were encouraged to switch to a process of planning their learning journey by instructing each participant to (1) discuss with someone in their group and identify how they could accomplish one or more of the pre-identified top five desired competencies; (2) list specific learning activities that could help using the CPD for Leaders Tool provided. The participants were encouraged to record their findings in the CPD for Leaders Tool and list one or two broad goals in addition to a few specific, measurable, achievable, relevant and timed (SMART) learning objectives for the areas of desired growth identified in an earlier exercise. Using the CPD for Leaders tool, participants were then instructed to identify some learning activities or resources that they could use to accomplish the pre-stated objectives. Additional information for consideration, such as specific experiential tasks (Table 2), were shared with the participants to provide ways in which leaders could further develop themselves. The activities listed were generated from experience. The last section of the CPD for Leaders workshop focused on evaluating the learning, which helps the participant to determine if they accomplished their intended objectives/goals and what task/activity they plan on doing next during their leadership journey. The facilitator provided opportunities for questions in the large group setting and concluded the workshop. The entire process lasted about 1 h.

After the Leadership Forum concluded, there were no suggestions for improvement received from AACP. The CPD for Leaders Tool was, however, updated to include two additional sections to benefit a longitudinal leadership development framework and online/virtual experience. The first section added allowed participants in a leadership development program to provide weekly or monthly reflection and journaling around leadership matters, challenges or learning encounters. Questions for the participant to consider include: What would you say you learned from the Leadership program this (Week/Month)? What did you start doing that is leadership related? What did you stop doing that is leadership related? What process or habit have you questioned that is leadership related? What are you proud of?

The second section added to the tool, provides the opportunity for feedback to the mentor or program organizer and can assist in program revision or refinement. Questions posed include: Overall, what would you say you have learned from the Leadership program this year? What went well with the program? What could we have done differently? What advice do you have for the next cohort of learners?

The updated CPD for Leaders Tool is ideal for leaders that might be interested in undergoing a longitudinal leadership development or mentoring program that builds upon some foundational leadership training or certificate course or workshop. The Tool was reintroduced to a group of approximately 30 young pharmacists who completed the Leadership Development Workshop at the 2019 International Pharmaceutical Federation (FIP) congress held in Abu Dhabi and its implementation is further described below [28]. The CPD tool has the potential to be used in many capacities, as well as incorporated into current and newly implemented leadership training programs for pharmacists wishing to improve their leadership skills. We describe in the next section some of the specifics that could assist in replicating the program in your organization.

## 4. Implementing an Online Leadership Development Program

The global leadership program was introduced in 2019 as a way to provide ongoing mentorship and further leadership development as young pharmacy leaders engaged with routine work activities at their home countries. Select leaders from the international pharmaceutical student’s federation (IPSF), Howard University College of Pharmacy and the FIP young pharmacists group (YPG) were a part of the team consulted to ensure that the program would be desirable to their colleagues. The main overarching goal in developing the program was to design a learning opportunity that could be replicated easily and would allow participants to deepen their learning obtained from a previously attended onsite leadership workshop. Another goal was to keep participants engaged throughout the year in a virtual environment while maximizing experiential opportunities in their home countries. Learning objectives for participants included helping them identify leadership development goals for the year, applying leadership concepts to accomplish established goals, implementing a leadership project using learned leadership concepts, and critiquing their personal progress in achieving their leadership goals. Young pharmacists concluding the Leadership Development Workshop at the FIP congress in Abu Dhabi were verbally introduced to the inaugural program. Interested participants signed up by sharing their name and email address with the facilitator after the workshop. The leadership content was proposed by one of the authors who is also a Global Lead for the Workforce Development Hub focusing on Leadership development (workforce development goal #6 or WDG6). The content and book chosen, had been used in prior leadership training sessions by this leader over the past decades. It was agreed to by the key stakeholders (IPSF, YPG, WDG6 Leads). Logistically, it was decided to hold one online meeting, via Zoom©, each month at a standardized time of GMT + 0 noon, to account for the varied time zones of all the participants. Meetings were 1.5 h and participants were required to have the ability to call in virtually to attend at least 80% of the sessions for a completion certificate at the end of the program. The program is designed for a virtual environment with an opportunity for completion in 10–12 months and allows for participation from around the world.

To begin the program, all participants used the CPD for Leaders Tool to identify gaps and areas of growth as well as the StrengthsFinder 2.0 online assessment to provide baseline self-awareness information [30]. All program participants were required to submit both the completed portions of the CPD tool and their top five strengths to confirm enrollment into the leadership program. During the first online session, session leaders reviewed the participants top five strengths results as a whole, to emphasize the unique leadership traits of the inaugural cohort. Throughout the duration of the 10-week program, participants were required to continually update their CPD tool using the reflection questions to track their leadership development.

Each monthly virtual meeting incorporated one chapter of the book, *Developing the Leader Within You* 2.0 by John Maxwell [31]. Between each meeting, program participants were asked to read the upcoming chapter, reflect on provided questions based on chapter content, and be ready to share at the next session. Each month’s session was led by one or two pharmacists from a team of international facilitators, each with a diverse range of interests (community, academia, research and administration) and backgrounds. The Leadership framework highlighting the key learning objectives, activities, program components, expectations, deliverables, and guidance for book discussants, are listed in Supplementary S2, while instructions to book lead discussants and a suggested online agenda is provided in Supplementary S3. Some of the lessons learned and opportunities for future leadership development are discussed in the following section.

## 5. Lessons Learned and Opportunities for Future Leadership Development

The submission of participant reflections on a periodic basis allowed program leads to evaluate the growth of the participants and the impact of the program. Thus far, two submissions, using the CPD for leaders Tool, have occurred. The first submission was in November 2019 and the second in January 2020. Participants answered questions about what they learned in the leadership program during the month, what they started doing that was leadership related, what they stopped doing that is leadership related, what leadership processes they had questions about, and what they are proud of achieving for themselves while in the program. In general, participants noted in their reflections that the program has helped them to increase awareness of their personal leadership strengths, has improved their leadership abilities by teaching them to provide validation to those they lead, improve time management and prioritization of tasks, improve people skills and understand the value of each member of the team, and as a leader know that it is okay to ask for help (Supplementary S4). Not only do participants reflect on what they are doing to improve their leadership abilities, but they have also shown growth in activities they stopped doing, reporting in their reflections that they are working to stop bad habits of micromanagement and working just to seek approval. Participants expressed that the program has helped them to grow as a leader and build their confidence to be able to lead. To date, participants in the program have expressed positive feedback and reflection about the leadership journey they are on with this program. No negative comments or feedback have been received yet about the program and its value.

From the facilitator’s standpoint, the sessions have gone well and were well received. We observed that the monthly sessions have helped the ongoing discussions on leadership topics and allowed participants to pose questions of interest to the facilitators, thereby opening up an informal mentoring opportunity for all participants. The authors plan to conduct a formal evaluation of the value of using the CPD for Leaders tool in addition to assessing the impact of the longitudinal program soon. Facilitators are making sure to be patient with participants who may have intermittent internet connectivity issues during a session.

There is much room for growth and development of leadership training programs for pharmacists. Currently, available programs often cater locally to participants of that country, and there are a limited number of programs that are available to an international audience. Leadership training programs also require out of pocket expenses from participants, many of which may choose not to participate due to financial reasons. Opportunities for sponsorship of such programs through grants could help facilitate participation from those who are unable to meet the financial obligations.

As leadership development programs are implemented, it is also vital to ensure that research surrounding their impact is pursued. Leadership skills can be developed in many ways and it is still unclear which methods and tools are the most effective in training pharmacists to maximize their leadership abilities. Additionally, research looking into how individuals who participate in leadership training programs compare to those individuals who do not, can shed further light on the value of such programs to the individual and their true value added, to the work of the pharmacy profession.

## 6. Conclusions

The importance of leadership development for pharmacists cannot be underestimated or overemphasized as an essential component to continuing professional development. As the roles of pharmacists continue to grow and young pharmacists explore opportunities in non-traditional pharmacy settings, leadership abilities are crucial to the success of not only the individual but also the profession. Programs and opportunities for pharmacists to advance their leadership skills should be available to all those who are interested. Examples of current programs that are available, as well as tools and implementation methods to develop novel, easily accessible, leadership development programs, are provided.

## Figures and Tables

**Table 1:** Select Leadership Development Opportunities Worldwide.

Asia	The Asian Young Pharmacist Group (AYPG) Leadership Summit consists of a panel discussion of four leaders who displayed exemplary leadership in their roles. The aim of the summit is to enhance the information shared between young Asian pharmacists and to increase the cooperation between members of the society. Leaders in the pharmacy profession from Malaysia, Indonesia, and the Philippines also share their leadership experiences at the summit. The participating countries consist of Japan, Singapore, Malaysia, Taiwan, Philippines, Cambodia and Indonesia (Thailand and Hong Kong via Video Call) [9].
Africa	The Pharmaceutical Leadership Development Program (PLDP) launched in South Africa in 2011. The aim of the development program was to strengthen management, leadership and governance skills in pharmacists and pharmacy managers. The development program is broken down into five workshops, taking place on a monthly basis. Participants are encouraged to apply the skills, information and other supporting tools to overcome the challenges faced in their workplace environment [10]. The Pharmacists Leadership Stimulant Program (PLSP) is a Nigerian program, aimed to develop and equip pharmacists with mentoring, coaching, employability and leadership training. The program consists of online courses, face-to-face meetings with mentors, peer-learning and additional tasks. The program is available for young pharmacists (below the age of 35) and final year pharmacy students [11].
North America	The Pharmacy Leadership & Education Institute (PLEI) is an educational foundation program, in the USA, dedicated to developing leaders in the pharmacy profession. The programs range from 1-h sessions to five-day programs. The PLEI also offers an in-depth workbook titled ‘Lead Grow Shape: A prescription for life-long leader development’ which includes several modules focusing on leadership. This is thought to be a customized leadership training program for all types of pharmacists [12]. The American College of Clinical Pharmacy (ACCP) Leadership and Management Certificate is an educational program that is designed to develop leadership and management skills in pharmacists. It is for those who already occupy leadership positions or aspire to such in the future. The program curriculum includes 18 h of core modules and 8 h of elective programming, totaling 26 h to complete the program. The core modules touch on topics such as the attributes of a leader, interpersonal and personal leadership development. However, the elective modules provide the participants with the opportunity to address specific leadership positions (e.g., pharmacy manager) or to apply a new leadership approach in practice [13]. The Pharmacy Leadership Academy (PLA) is an online program which is made up of seven courses. The course topics are built upon a set of acknowledged skills that are thought to be required in an effective leader. Examples of courses include: Leadership Influence, Leading Effective Financial Management and Strategic Clinical Leadership. A different amount of time is needed to complete each individual course, but the majority of them can be accomplished in a month [14].
Europe	The Top Pharmacy Leadership program by the Centre of Pharmacy Postgraduate Education (CPPE) was delivered alongside the NHS Leadership Academy (an academy providing leadership development opportunities for healthcare workers) to equip aspiring or new-in-role senior pharmacists with the appropriate skills needed for them to carry out their leadership position. However, due to the withdrawal of NHS funding, the program will not be available in 2020 [8]. The CPPE Leading for Change program is a program for mid-to-senior pharmacy professionals who are working at, or towards, an advanced level of practice. The program takes place over six months and is a blend of face-to-face workshops, online modules, and work-place based learning experiences. The aim of the program is to develop leaders throughout the pharmacy profession, resulting in better patient outcomes [15]. Online self-assessment tools are also available. The NHS network has provided NHS workers with an online tool to identify their leadership strengths and weaknesses. This was designed to assist with their professional development [16]. The Clinical Leadership in Pharmacy program accredited by the Royal Pharmaceutical Society is available for primary care pharmacists and hosted by the Primary Care Pharmacy Association (PCPA). The aim of the program is to “unlock the potential for future pharmacy leaders”. It had previously been run by pharmacists in Scotland and was introduced to England in 2018 [17]. Health Education England (HEE) offers a range of programs for community pharmacists and pharmacy technicians to build upon and enhance their leadership skills. Community pharmacists are encouraged to take advantage of the fully funded NHS training program. It is an online program with minimal face-to-face contact; therefore, pharmacists are able to engage in this program in their free time. Suitable for those who require flexibility, they are delivered through universities across the country [18]. Mary Seacole Leadership Programmes for Community Pharmacists – With investment from the Pharmacy Integration Fund (PhIF), this programme is delivered as a 6-month leadership programme in London, Leeds and Birmingham. There are eight units on leadership fundamentals, and this is also accompanied by management skill training. This programme is under the NHS Leadership Academy [19]. In Scotland, A Taste of Leadership (TOL) is a foundational one-day course open to all pharmacists in their early career. This day course takes place in February and September. It aims to teach pharmacy professionals about leadership fundamentals such as emotional intelligence, teamwork and personal awareness [20]. In Wales and Ireland, there is a Pharmacy Clinical Leadership Programme (CLIP) available for future pharmacy leaders. It is a comprehensive leadership series which includes a broad range of training events. The programme itself consists of 10 interactive learnings days spread over 12 months. The participants are also provided with written material to benefit the learners [21,22].
Australia	The University of Sydney introduced a new program to the pharmacy course for the university students at Sydney University, giving students the opportunity to develop their leadership skills outside of a class setting. It aims to develop students to be influential global leaders. It will be separate from the pharmacy degree, with students engaging in online modules and seminars in a course to graduate from the leadership program [23]. The Pharmaceutical Society of Australia has a 12-month leadership program which offers highly interactive workshops that focus on leadership, communication, networking and many more. The program aims to help pharmacists realize their leadership potential. It is for early-career pharmacists (less than 10 years post-graduation) [24]. The Society of Hospital Pharmacists of Australia (SHPA) introduced a seven-day intensive pharmacy leadership program to North America. This national society is focused on the continued development of their members, with a strong focus on the promotion of excellence in medicines management. In 2019, the program was one-week long. It is a 10-person program where nine spaces are available for hospital pharmacist leaders and one space allocated for an “emerging” pharmacist [25]. The Leaders Evolving and Developing (L.E.A.D) Program is a one-day bootcamp course to help pharmacists develop skills that staff in the pharmacy profession must possess to be effective leaders in the workplace [26]. The program will include teachings on: the skills and strengths required to construct a team capable of meeting demands, pharmacy finance and accountability in leadership success and strategic planning.
Global	The Leaders in Training (LIT) workshop is hosted by the International Pharmaceutical Students Federation and aims to equip the future pharmacy workforce with the necessary leadership and management skills. Select topics covered include time and project management, public speaking and communication, and networking with future pharmacy leaders [27]. The Young Pharmacist Group Leadership Development Workshop is hosted by the International Pharmaceutical Federation (FIP), a global body representing over four million pharmacists and pharmaceutical scientists. The workshop is available annually for young pharmacists who are interested in growing in leadership. One of the aims is to develop soft and practical skills in leadership and management. International speakers focus on leadership styles, understanding personalities within the workplace, project management, communication, strategic planning and servant leadership [28].

**Table 2:** Experiential Tasks to Consider for Leadership Development.

Attending meetings Leading a meeting Giving presentations Solving problems instead of delegating up Advising student organizations Listening to and discussing with other leaders Working on projects with a timeline Establishing a research lab or clinical site Managing startup funds, budgets and personnel Implementing major change Managing change initiatives

## Supplementary Materials

The following are available online at https://www.mdpi.com/2226-4787/8/2/79/s1, Supplementary S1: Continuing Professional Development (CPD) for Leaders Worksheet, Supplementary S2: Longitudinal Leadership Development Framework, Supplementary S3: Instructions to Book Discussants and Suggested Agenda, Supplementary S4: Sample reflections from participants on leadership development and growth during the program.
