# Supplementary material for: Implementing an Online Longitudinal Leadership Development Program Using a Leadership-Specific Continuing Professional Development (CPD) Tool
Source: Pharmacy (Basel). 2020 May 6;8(2):79. doi: 10.3390/pharmacy8020079 (PMC7356594; doi:10.3390/pharmacy8020079)
Supplement: Supplementary file 1 [file pharmacy-08-00079-s001.pdf]

Supplementary S1. Continuing Professional Development (CPD) for Leaders Worksheet

Continuing Professional Development (CPD) for Leaders Worksheet
A.K.A. CPD for Leaders

REFLECT

Individual and Peer Reflection:

List the desired attributes of a Leader

1. ______________________________
2.
3. ______________________________
4.
5. _______

REFLECT

Personal Reflection: Leader CPD

List YOUR desired competencies as a Leader

1. ______________________________
2.
3. ______________________________
4.
5. ______________________________

PLAN

State 1-2 broad goals associated with your desired competencies:

Create one or more SMART* learning objective(s) for the areas you have identified:

***Definition of SMART:**

S-Specific: state precisely what you will be able to do
M-Measurable: include observable or quantifiable action/behavior
A-Achievable: integrate action that is feasible/realistic
R-Relevant: align with practice, administrative and/or organizational goals T-Timed: include a target date to be completed

Format example:

By January 20xx, I will develop a medication education program for children and adolescents with ADHD receiving services at the community mental health clinic where I practice.

LEARN
Identify some **learning activities/resources** that you will engage in to meet your objective(s). (Select all that apply)

Continuing Education

o CE activities by ACPE accredited providers

o CE activities by accredited providers from other disciplines

Academic/Professional Study

o Participating in conferences, workshops

o Undertaking academic coursework or postgraduate education
o Completing certification courses
o Leading or participating in journal/book clubs
o Other

Scholarly Activities

o Leading a group of researchers in one's professional field
o Presenting/publishing scholarly works

Teaching and Precepting

o Mentoring others

o Peer coaching or mentoring
o Other

Workplace Activities

o Engaging in Point-of-decision learning

o Training to learn a new skill

o Leading improvement projects

o Consultation with peers/healthcare professionals to address specific issue
o Serving on committees
o Other

Professional/Community Service

o Active involvement in professional associations/organizations
o Volunteer work

o Other

Other ____
____

For more on CPD activities: https://www.acpe-accredit.org/pdf/CPDGuidance%20ProfessionPharmacyJan2015.pdf

EVALUATE/APPLY

Considering the learning activity/activities you completed did your knowledge/abilities: (**select best option**) ______________________

Stay the same

Improve somewhat, but I was hoping to learn more

Improve somewhat, and I am satisfied with my knowledge/ability

Improve greatly, but I still want to learn more

Improve greatly, and I am satisfied with my knowledge**/ability**

What is the outcome of your learning activity/activities? (select best option) No change/reinforced my current knowledge/professional habits.

I implemented what I learned.

Barriers prevented implementation.

- Lack of time
- Lack of resources (e.g., financial, technology, personnel)
- Systems-related
- Changed positions/practice
- Other____

In progress

- More information or learning is needed before I can implement in practice.
- Modification of goals/objectives.
- Additional reflection on professional development needs.
- Other____

If implementation of learning occurred, how did it impact your leadership? (select all that apply)

Improved my confidence

Shared information with supervisor/colleagues

Addressed organization specific concern/need

Modified existing services/practices

Improved processes/culture

Other:____ Not Applicable (implementation did not occur)

(YOU MAY DUPLICATE THIS PAGE TO ALLOW CONTINUAL REFLECTION THROUGHOUT THE PROGRAM)

What would you say you learned from the Leadership program this (Week/Month)?

What did you start doing that is leadership related?

What did you stop doing that is leadership related?

What process or habit have you questioned that is leadership related?

What are you proud of?

RECORD: List of CPD Learning Activities

Date	SMART Objective	Learning Activity	Outcome

After completion of the leadership program, please complete the following and send to the Mentor/organizer:

Overall what would you say you have learned from the Leadership program this year

What went well with the program?

What could have been done differently?

What advice do you have for the next cohort of learners?

Supplementary S2. Longitudinal Leadership Development Framework

Leadership Development Framework

Main Outcomes

1. Apply the key leadership principles obtained during a prior Leadership Development program/workshop/certificate. (Teamwork, project management, communication, leadership, continuing professional development)

2. Engage participants in the organization work/system.

Learning Objectives

At the completion of this program, participants should be able to:

1. Identify leadership development goals for the year.
2. Apply learned leadership concepts towards accomplishing your personal leadership goals
3. Implement a leadership project to work on with a team
4. Critique personal progress towards identified leadership development goals

Deepen the learning activities

1. Complete the Leadership CPD profile at the beginning
2. Read Strengthsfinder 2.0
3. Complete the Strengths finder assessment and identify your top 5 strengths
4. Present findings from a project worked on during the year with your team
5. Provide feedback on how to strengthen the leadership development experience

4. Developing the leader within you 2.0- John MAXWELL Required book chapter discussion monthly during the year. 1-1.5-hour book discussion. Different leads could run the sessions each month. Program runs longitudinally over a year

5. Work on projects. Leaders or facilitators can share a potential list of projects that they can be involved in. (description, timelines, deliverables etc.)
6. Participants update the leadership CPD portfolio, monthly, midpoint and at the end of the year and submit to the organizers. (lessons learned, challenges, resources used etc.)
7. Teams seek an opportunity to present learnings around. 1.) Leadership lessons learned or applied during the year. 2.) Accomplishments in projects worked on during the year.

Program Components:

- Completion of CPD portfolio
- Completion of strengths finder test to determine top 5 strengths
- Monthly Book Discussions- Book Title **Developing the Leader Within You 2.0** by John Maxwell
- Monthly leadership reflections – to be reviewed by each chapter book lead discussant/co-lead

Participant Expectations and Deliverables

1. Attend the information session scheduled in October

2. Complete the following and submit to the Leadership 2.0 Program Coordinators **by November 1st**

    1. Purchase a copy of the Strengths finder test/book

    2. Complete the test, write down your top 5 strengths and download your report for future personal use and action

    3. Sending the results will be the indication of investment and commitment to being part of the pilot program

3. Purchase a copy of the book and **read chapter one before the first meeting on November 13th**. We strongly encourage you to obtain the workbook version to use throughout the year.

4. Complete the first 1-4 pages of the **CPD Leadership Portfolio** and submit **by the first session.**

5. Review emails regularly and follow ALL instructions

6. Attend and actively participate in at least 8/10 of the **monthly book chapter discussion** sessions.
    1. The Definition of Leadership
    2. The Key to Leadership: Priorities
    3. The Foundation of Leadership: Character
    4. The Ultimate Test of Leadership: Creating Positive Change
    5. The Quickest Way to Gain Leadership: Problem Solving
    6. The Extra Plus in Leadership: Attitude
    7. The Heart of Leadership: Serving People
    8. The Indispensable Quality of Leadership: Vision
    9. The Price Tag of Leadership: Self-Discipline
    10. The Expansion of Leadership: Personal Growth
    11. Wrap up longitudinal program and review Final CPD Portfolio

7. Complete periodic/monthly **leadership reflections** on your journey and learnings

8. Complete the final CPD leadership Portfolio **by program end in August 30th**

9. Participate as a leader in a project.

Supplementary S3. Instructions to Book Discussants and Suggested Agenda

**Instructions to Book Discussants**

1. Please consider identifying discussion questions to share with the class ahead of time so that they can be ready to discuss with you. Your workbook has discussion questions at end of your assigned topic/chapter that you can use.

2. The organizer will be sending the virtual meeting invitation for your assigned month to you. This will include the names of all the participants in the program.

3. Please be aware that they have all attended the leadership development program at Abu Dhabi. See topics received in the following link. https://abudhabi2019.fip.org/programme-session/young-pharmacists-group-leadership-development-workshop-part-1-d6/

4. Since it is a Zoom call, you are strongly encouraged to engage all the participants on the call so they don't tune out during the time with you. Use of video may reduce the quality of the call.

5. We have used the audio portion of the call so far with good success. Please note that some participants in LMICs may have connectivity issues and drop in and out during the session.

Suggested Agenda for the Monthly Online sessions

1. Welcome and introductions
2. Leadership Reflections of the month since last meeting
3. What did you learn? Use some of the specific questions in the workbook to guide the conversation.
4. What surprised you?
5. Share a personal experience on the topic and how it helped you grow as a leader
6. What will you do differently after this call?
7. Wrap up and announce the next call. Direct concerns to the organizers.

Supplementary S4: Sample reflections from participants on leadership development and growth during the program

1. **What would you say you learned from the Leadership program this (Week/Month)?**
    - I learnt how I should focus on my strengths and use it in teamwork and in strategic thinking, executing, influencing projects while building relationships. I also learnt about the 5 level of leadership where I identified myself as currently a positional leader.
    - I have learned about giving validation to people. On the foundation of leadership which is character, I learnt that my words should always match with my actions. I have always known this but during the discussion, it made more sense to me how not doing this leads to gradual loss of trust and integrity. I also learnt on how to communicate especially when you do not have 100% control of the outcome
    - The importance of prioritizing tasks and using tools such as the Pareto Principle, the Three 'Rs' and making room for margin, to achieve this.
    - I learned that we need to be proactive about planning out time and that if we do not prioritize properly we would find ourselves giving room for others' tasks to the detriment of ours
    - I learned the importance of character and self-leadership.
    - How far you can travel isn't the point; it's how far you are able to take your people.
    - People buy into the leader, then the vision.
    - The importance of having several mentors, the effect of personal integrity on producing results in leadership, the process of validating others
    - I learnt the importance of NO in setting priorities.
2. **What did you start doing that is leadership related?**
    - I started to be more conscious about building relationships with my team members.
    - Revisited all the promises….and started making deliberate step to act on it
    - Working actively to improve my decision-making skills my being proactive and taking initiative
    - More effective time management
    - Actively delegating tasks to my team and understanding where to draw the limit between what I have to do myself and what I have to delegate.
    - Re-evaluating the goals I set for myself and my team and analyzing where I may have fallen short, and explaining to them when I cannot meet up to what I said, with valid explanations and a more certain plan
    - Communicate and reinforce. Attend to the worst fears first
    - Intentionally interacting more with my colleagues
    - I created a work plan where I only choose two priorities to focus on; I divided them into larger goals and short term in order to lead my team better.
3. **What are you proud of?**
    - Being a part of this LDP and continuing so far. I usually give up so easily and do not have very much patience but I am proud of myself being here and engaging on all the activities so far
    - I am proud that I am now being more positive and proactive with my team members
    - My increased self-awareness and confidence to take charge of tasks and projects, albeit doing it mostly afraid
    - That I have actually in the recent practiced a relatively effective method of prioritization with good results. Also that I am becoming more confident to delegate tasks and provide instructions.
    - My increased confidence as a leader and my improved ability to guide team discussions in a meeting
    - My openness to change and the courage I have had to initiate change repeatedly
    - I am now more proactive in building relationships
    - More focus and direction in how I utilize my time and resources.
